# Hypnotism in Surgery

**Published:** 1895-10

**Authors:** 


HYPNOTISM IN SURGERY.—Fifty years ago Dr. Dugus operated several times upon a woman in the hypnotic state. The patient gave no indication whatever of sensibility. He believed that the phenomena of "mesmeric sleep" might be so utilized as to render it available to all sufferers in surgical cases. A little earlier Dr. Long, one of the discoverers of anæsthesia, had investigated the subject, but did not use hypnotism because of its supposed uncertainty of action. Hypnotism has thrice been revived and twice fallen into disuse during the last century.

